# Controlling Microbial Safety Challenges of Meat Using High Voltage Atmospheric Cold Plasma

**DOI:** 10.3389/fmicb.2016.00977

**Published:** 2016-06-22

**Authors:** Lu Han, Dana Ziuzina, Caitlin Heslin, Daniela Boehm, Apurva Patange, David M. Sango, Vasilis P. Valdramidis, Patrick J. Cullen, Paula Bourke

**Affiliations:** ^1^School of Food Science and Environmental Health, Dublin Institute of TechnologyDublin, Ireland; ^2^Department of Food Studies and Environmental Health, Faculty of Health Sciences, University of MaltaMsida, Malta; ^3^School of Chemical Engineering, University of New South WalesSydney, NSW, Australia

**Keywords:** high voltage plasma, biofilm, beef extract, ROS, Gram negative and positive bacteria

## Abstract

Atmospheric cold plasma (ACP) is a non-thermal technology, effective against a wide range of pathogenic microorganisms. Inactivation efficacy results from plasma generated reactive species. These may interact with any organic components in a test matrix including the target microorganism, thus food components may exert a protective effect against the antimicrobial mode of action. The effect of an in-package high voltage ACP process applied in conjunction with common meat processing MAP gas compositions as well as bacteria type and meat model media composition have been investigated to determine the applicability of this technology for decontamination of safety challenges associated with meat products. *E. coli, L. monocytogenes*, and *S. aureus* in PBS were undetectable after 60 s of treatment at 80 kV_RMS_ in air, while ACP treatment of the contaminated meat model required post-treatment refrigeration to retain antimicrobial effect. The nutritive components in the meat model exerted a protective effect during treatment, where 300 s ACP exposure yielded a maximum reduction of 1.5 log using a high oxygen atmosphere, whilst using air and high nitrogen atmospheres yielded lower antimicrobial efficacy. Furthermore, an ROS assay was performed to understand the protective effects observed using the meat model. This revealed that nutritive components inhibited penetration of ROS into bacterial cells. This knowledge can assist the optimization of meat decontamination using ACP technology where interactions with all components of the food matrix require evaluation.

## Introduction

In the food production chain, meat safety remains a major industrial challenge presented by the emergence of pathogens with low infectious doses, increased virulence, resistance to antibiotics and cross-contamination or recontamination of foods, food contact surfaces and water (Sofos and Geornaras, 2010). Efficient strategies are required to reduce the microbiological safety risks of meat products in tandem with the goal of shelf-life extension.

Modified atmosphere packaging (MAP) is widely used in the food industry to extend shelf-life and avoid contamination and weight loss (Sivertsvik et al., 2002; Kerry et al., 2006). Nitrogen is the most widely used gas in MAP, as an inert filler gas either to reduce the proportions of the other gases or to maintain pack shape (Kerry et al., 2006). High oxygen levels (70–80%) are also used in MAP to reduce microbial growth within the package and preserve the bright red color of fresh meat as well as tenderness and juiciness (Okayama et al., 1995; Lund et al., 2007). Carbon dioxide is also employed for inhibiting bacterial growth (Sivertsvik et al., 2002), and maintaining the red color of meat products. Typically, fresh red meat MAP uses 70% O_2_+30% CO_2_ (Sørheim et al., 1999) and cooked meats are stored in 70% N_2_ + 30% CO_2_ (Smiddy et al., 2002).

The efficacy of atmospheric cold plasma (ACP) has been documented for microbial inactivation in a number of food products and the underlying mechanisms of the bactericidal effect have been demonstrated to result partly from cell-lethal reactive species generation (Kayes et al., 2007; Basaran et al., 2008; Klämpfl et al., 2012; Rød et al., 2012; Misra et al., 2014; Ziuzina et al., 2014; Lacombe et al., 2015; Yong et al., 2015). With the emerging range of ACP applications and complexity of system design available, it is important to consider the mechanism of action in relation to critical processing parameters. By discharging in air, the reactive species generated include reactive oxygen species (ROS), reactive nitrogen species (RNS), ultraviolet (UV) radiation, energetic ions and charged particles. ROS are a principal reactive group of species with relatively long half-lives and strong anti-microbial effects (Joshi et al., 2011). These reactive species are believed to cause damage to proteins and nucleic acids and lesions to the cell membrane (Laroussi et al., 2003). However, a number of experimental parameters have been shown to influence the bactericidal efficiency of ACP governing the type and amount of reactive species generated, such as applied voltage (Yun et al., 2010) and treatment time (Miao and Yun, 2011; Ziuzina et al., 2014). Bacterial inactivation also varies depending on the type of bacteria (Montie et al., 2000; Yu et al., 2006; Fernández and Thompson, 2012). Therefore, in the current study, three strains were selected to represent Gram negative and Gram positive bacteria, with bacterial resistance and industrial relevance also considered (Le Loir et al., 2003; Doyle and Erickson, 2006; Denny and McLauchlin, 2008). *E. coli* NCTC 12900 and *S. aureus* ATCC 25923 are key food-contaminating pathogens, and have been reported to have strong multi-drug resistance (Brown, 2001; Braoudaki and Hilton, 2004). Their high rate of mutations can lead to cross protective effects against environmental stresses, including oxidative stress. *L. monocytogenes* has the capability to reproduce at low temperatures with the regulation of cold shock proteins (Bayles et al., 1996), making it a major pathogen of concern in the meat cold chain. Moreover, *L. monocytogenes* has been shown to be the most resistant on meat surfaces compared with the other key food pathogens (Chorianopoulos, 2012). As many contaminating pathogens in the food processing environment present in resistant biofilm structures attached to biotic or abiotic surfaces, it is important to assess the efficacy of novel decontamination methods against the actual nature of the microbiological challenges presented, thus critically evaluating the potential to reduce risks. Biofilms and attached cells are characterized by an enhanced resistance when compared to their planktonic counterparts for most environmental stresses encountered in food production plants (Giaouris et al., 2014; Bridier et al., 2015).

Although ACP technology is increasingly studied for food decontamination, there are limited studies pertaining to its application on meat products. Therefore, this study provides a detailed investigation of the bactericidal effect of ACP on three key meat-contaminating pathogens using a complex meat model media and elucidates the role of ROS in the disinfection process of ACP. Industry-relevant processing parameters that could interact with ACP efficacy and the mode of action were evaluated by using typical MAP gases for meat products as plasma working gases as well as storage temperature. Therefore, these interactive effects were assessed with regard to ACP efficacy in a meat model to provide insight for the application of ACP for meat decontamination.

## Materials and methods

### Experiment preparation

#### Bacterial strains and growth conditions

*Listeria monocytogenes* NCTC 11994 and *Staphylococcus aureus* NCTC 1803, were obtained from the microbiology stock culture of the School of Food Science and Environmental Health, Dublin Institute of Technology. *Escherichia coli* NCTC 12900, (non-toxigenic O157:H7) was obtained from the National Collection of Type Cultures of the Health Protection Agency (HPA, UK). Strains were selected to present both Gram positive and Gram negative foodborne challenges and to facilitate comparison with other studies. Strains were maintained as frozen stocks at −70°C in the form of protective beads, which were plated onto tryptic soy agar (TSA, Scharlau Chemie, Barcelona, Spain) and incubated overnight at 37°C to obtain single colonies before storage at 4°C.

#### Preparation of planktonic bacterial cell suspensions

Cells were grown overnight (18 h) by inoculating an isolated colony of each bacteria in 12% Beef Extract (BE, Scharlau Chemie, Barcelona, Spain) at 37°C. Cells were harvested by centrifugation at 10,000 rpm for 10 min. The cell pellet was washed twice with sterile phosphate buffered saline (PBS, Oxoid LTD, UK). The pellet was re-suspended in PBS and the bacterial density was determined by measuring the absorbance at 550 nm using McFarland standard (BioMérieux, Marcy-l'Étoile, France). Finally, cell suspensions with a concentration of 10^7^ CFU ml^−1^ were prepared in PBS, 3 or 12% BE, and then used for inactivation of bacteria in planktonic state and ROS studies. Prior to ACP treatment, aliquots of 100 μl per well cell suspensions in PBS, 3 or 12% BE were dispensed into the wells of a microtiter plate for planktonic samples.

#### Biofilm formation

Bacterial biofilms were produced by adding 200 μl of 12% BE bacterial suspension with a cell density of 1.0 × 10^7^ CFU ml^−1^ into the wells of 96 well microtiter plates (Sarstedt, Nümbrecht, Germany).

The plates were incubated at 37°C for 48 h. After 24 h of incubation the supernatant from each well was replaced with fresh BE, with further incubation for 24 h. After incubation, the BE containing the suspended bacterial cells was removed and wells rinsed three times with sterile deionized water and dried, leaving only bacterial biofilms for further investigation. Negative controls were obtained by using BE without inoculation. Prior to each experiment, the microtiter plates containing biofilms were air dried for 60 min.

#### ACP system configuration

The DBD ACP system used in this study has been described by Han et al. (2015) and its electrical properties has been reported by Moiseev et al. (2014). The system was operated at high voltage levels of 60, 70, and 80 kV_RMS_ under atmospheric pressure. A polypropylene container, which acted as a sample holder and a dielectric barrier, was placed between the two perspex dielectric layers. The overall distance between the two electrodes was kept constant (3 cm) for all experiments.

### ACP treatment time, voltage level, and post-treatment storage

Each microtiter plate was placed in the center of the rigid polypropylene plastic container, which was positioned directly between the electrodes within the plasma discharge for direct plasma treatment. The distance between the sample and top electrode was 20 mm. Each container was sealed with a high barrier polypropylene bag (Cryovac, B2630, USA). Air (gas 2) was used as the working gas for generation of ACP. For the gas mixtures of 30% CO_2_ + 70% N_2_ (gas 1) and 30% CO_2_ + 70% O_2_ (gas 3), the required working gas was filled into a sealed and pre-vacuumed package using a flow regulator at a controlled flow rate of 0.5 L min^−1^ for 1 min. To assess the effect of voltage on inactivation efficiency, bacterial samples were treated with either 60, 70, or 80 kV_RMS_, over a range of treatment times (60, 120, or 300 s). Bacterial samples in PBS were exposed to shorter treatment times (15, 30, or 60 s). After treatment, samples were stored at either room temperature or 4°C for 24 h to assess the effect of ACP generated reactive species during post-treatment storage time (Han et al., 2014). All experiments were carried out in duplicate and replicated twice.

### Post-treatment analysis

#### Ozone measurements

Ozone concentrations generated inside the sealed packages were measured using GASTEC gas tube detectors (Product # 18M, Gastec Corporation, Kanagawa, Japan) immediately after treatment and also after 24 h storage.

#### Detection of reactive oxygen species after ACP treatment

DCFH-DA (2′,7′-dichlorodihydrofluorescein diacetate) is a cellular assay probe widely used for fluorescence detection of intracellular ROS (Gomes et al., 2005).

After ACP treatment and storage, cells were incubated with DCFH-DA (Sigma-Aldrich, USA) at a final concentration of 5 μM in PBS for 15 min at 37°C. Two hundred micro liter aliquots of each sample were transferred into 96 well fluorescence microplate wells (Fisher Scientific, UK) and measured by a Synergy™ HT Multi-Mode Microplate Reader (BioTek Instruments Inc.) at excitation and emission wave lengths of 485 and 525 nm (Joshi et al., 2011). However, extracellular reactive species may have interference and result in higher fluorescence signals (Gomes et al., 2005). The results represent total ROS effect after discharging. Fluorescence data were obtained by subtracting the background fluorescence of the respective untreated control cells, due to the much higher background from BE than PBS. These data represent the ROS concentrations generated after ACP treatment expressed as arbitrary fluorescence units (AFU).

#### Microbiological analysis

To assess effects on planktonic bacterial populations, dilutions were prepared using maximum recovery diluent (MRD, Scharlau Chemie, Barcelona, Spain), which were further plated on TSA. In order to obtain low microbial detection limits, 1 ml and 0.1 ml of the treated sample was spread onto TSA plates as described by EN ISO 11290-2 method (ISO 11290-2, 1998). The limit of detection was 1.0 log_10_ CFU ml^−1^. Plates were incubated at 37°C for 24 h and colony forming units were counted. Any plates with no growth were incubated for up to 72 h and checked for the presence of colonies every 24 h. Results are reported in log_10_ CFU ml^−1^ units.

To quantify the effects of ACP treatment on biofilms, PBS (100 μl) was added into the wells containing biofilms. The 96 well plate was then sonicated for 5 min to detach the bacterial cells into the solution using a water bath sonicator (Bransonic 5510E-MT, USA, Mexico). PBS cell suspensions from each well for corresponding samples were mixed to obtain an average surviving bacterial population, serially diluted in MRD and surface plated on TSA.

#### Modeling microbial recovery

Two hundred micro liter aliquots of bacterial suspensions in 3% BE were pipetted into 96 well microtiter plates. The initial cell density was 1.0 × 10^7^ CFU ml^−1^ with four decimal dilutions of the suspensions performed in the microtiter plate. Samples were ACP treated for 60, 120, and 300 s and stored at 4°C for 24 h post-treatment. Twenty micro liter aliquots from each well were analyzed using plate count. The microtiter plates were then placed in a microplate spectrophotometer (ELx808, BioTek Instruments Inc., U.S.A.) set at 37°C. Absorbance readings were taken at 600 nm every 30 min for 48 h. The OD values were used to quantify the growth rate, μ_*max*_ and the lag phase, λ, of all the bacteria studies using the following equation (Cuppers and Smelt, 1993; Biesta-Peters et al., 2010):

(1) $$\begin{array}{l} {TTD}_{i}=\lambda \;+\;\frac{1}{{\text{μ}}_{max}}\cdot log\left(\frac{N_{turb}}{N_{i}}\right) \end{array}$$

where *TTD*_*i*_, is time to detection (h) of the inoculum level *i*, chosen as the time at which the sample in the well reaches an OD_600_ of 0.2 (Biesta-Peters et al., 2010), λ is the duration of the lag phase (h), *N*_*turb*_ is the number of bacteria per ml (CFU ml^−1^) at which an OD_600_ of 0.2 is observed, *N*_*i*_ is the number of organisms per ml of the inoculum at time zero, and μ_*max*_ is the maximum specific growth rate (h^−1^). *N*_*turb*_ was determined through separate experiments. The goodness of fit of these curves was validated experimentally. The kinetic parameters (μ_*max*_ and λ) were calculated by performing a regression analysis on the data correlating *TTD* with log (*N*_*turb*_/*N*_0_) using equation 1 for each replicate separately (Millan Sango et al., 2015).

### Statistical analysis

Results were analyzed using SPSS 22.0 (SPSS Inc., Chicago, U.S.A.). Means were compared using analysis of variance (ANOVA) using Fisher's Least Significant Difference-LSD at the 0.05 level.

## Results

### Effect of applied voltage on ACP inactivation efficacy

**Figure 1:**
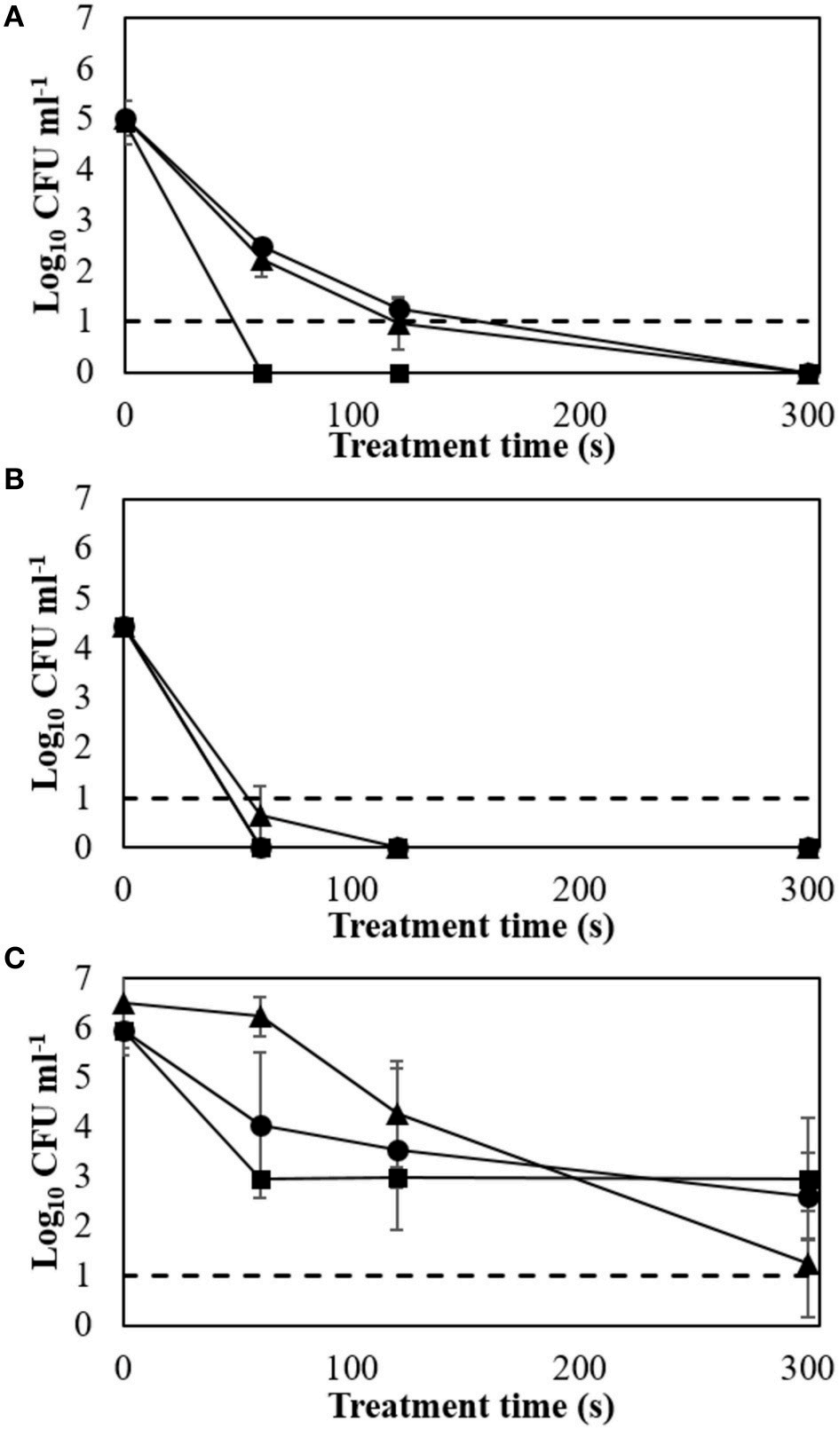
**Biofilm inactivation efficacy with applied voltage and treatment time (24 h storage at room temperature). (A)** *E. coli* NCTC 12900; **(B)** *L. monocytogenes* NCTC 11994; **(C)** *S. aureus* NCTC 1803. Dotted line: detection limit; ▲: 60 kV_RMS_; ●: 70 kV_RMS_; ■: 80 kV_RMS_.

The effect of a range of high voltage levels (60, 70, and 80 kV_RMS_) on ACP efficacy against key meat pathogens grown as biofilms using a meat model medium was investigated (Figure 1).

Treatment at 60 or 70 kV_RMS_ had similar inactivation effects for *E. coli* with approximately 2 log cycles remaining after 60 s of ACP exposure. However, complete inactivation was obtained after 60 s of treatment at 80 kV_RMS_ (Figure 1A). *L. monocytogenes* biofilm showed greater susceptibility, where similar inactivation patterns were found for 60, 70, and 80 kV_RMS_ treatment, with non-detectable cell concentrations after 60 s (Figure 1B).

Extending treatment time from 120 to 300 s at 60 and 70 kV_RMS_ reduced *S. aureus* biofilms significantly. After 60 s treatment at 80 kV_RMS_, bacterial concentrations were reduced to 2.96 ± 0.14 log cycles. However, extending the exposure time at 80 kV_RMS_ did not lead to further significant reductions in population density (Figure 1C).

**Figure 2:**
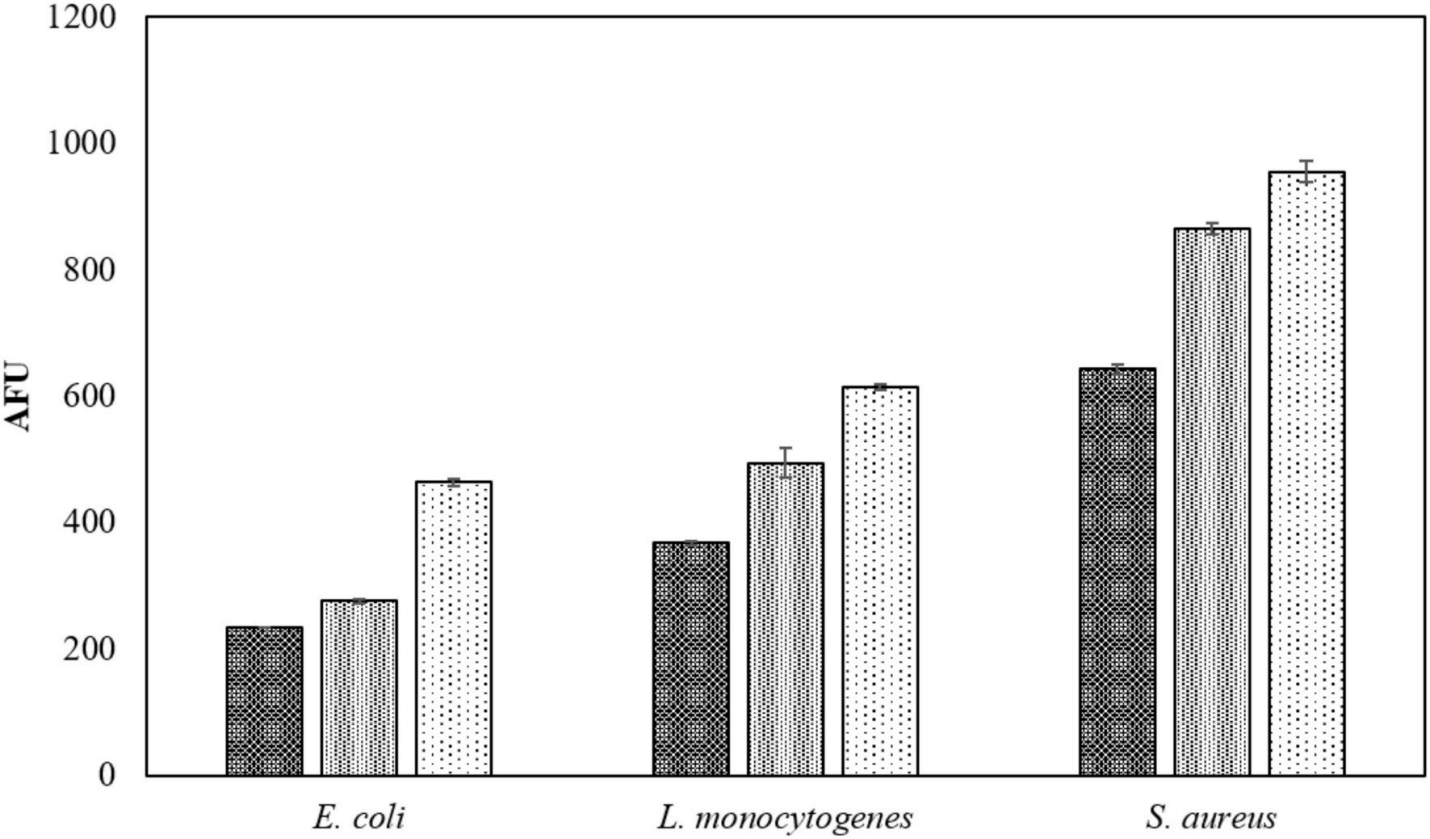
**Voltage associated intracellular ROS density assay by DCFH DA**. Samples in PBS were treated for 60 s at 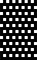 60 kV_RMS_, 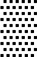 70 kV_RMS_, and 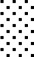 80 kV_RMS_ and analyzed without post-treatment storage.

The fluorescent probe used for measuring ROS is more accurate when using planktonic cells in PBS and thus the positive effect of voltage on the ROS densities in liquid samples is shown in Figure 2. Fluorescence levels gradually increased with applied voltages of 60, 70, and 80 kV_RMS_.

### Effect of media and post-treatment storage temperature on ACP inactivation efficacy

Tables 1–3 show the inactivation efficacy of ACP treatment at 80 kV_RMS_ against planktonic cells of *E. coli, L. monocytogenes*, and *S. aureus*, respectively, as a function of treatment time, media composition, and storage temperature conditions.

**Table 1:** *****E. coli*** NCTC12900 planktonic inactivation efficacy at 80 kV_**RMS**_ with different media and post-treatment storage conditions**.

**Media**	**Plasma treatment time (s)**	**Storage condition**			
		**4**°**C storage**		**RT storage**	
		**Cell density (Log_10_ CFU ml^−1^)**	SD^*^	**Cell density (Log_10_ CFU ml^−1^)**	SD^*^
3% BE	C^1*^	7.11^ab^	0.06	6.96^a^	0.19
	C^2*^	6.95^bc^	0.11	9.18^b^	0.16
	60	7.35^b^	0.25	9.11^b^	0.13
	120	6.73^c^	0.13	9.15^b^	0.13
	300	6.06^d^	0.15	8.41^c^	0.55
12% BE	C^1*^	7.13^ab^	0.16	7.02^a^	0.29
	C^2*^	7.18^ab^	0.21	9.39^d^	0.07
	60	7.32^b^	0.04	9.47^d^	0.17
	120	7.12^c^	0.68	9.33^bd^	0.26
	300	5.79^d^	0.92	9.35^d^	0.36
PBS	C^1*^	7.18^ab^	0.09	6.94^a^	0.19
	C^2*^	6.94^bc^	0.09	6.90^a^	0.00
	60	ND^*e^	–	ND^*e^	–
	120	ND^*e^	–	ND^*e^	–
	300	ND^*e^	–	ND^*e^	–

SD^*^, standard deviation; ND^*^, non-detectable; C^1*^, Control without storage; C^2*^, Control with storage. Different letters indicate a significant difference at the 0.05 level between media and treatment times.

**Table 2:** *****L. monocytogenes*** NCTC11994 planktonic inactivation efficacy at 80 kV_**RMS**_ with different media and post-treatment storage conditions**.

**Media**	**Plasma treatment time (s)**	**Storage condition**			
		**4**°**C storage**		**RT storage**	
		**Cell density (Log_10_ CFU ml^−1^)**	SD^*^	**Cell density (Log_10_ CFU ml^−1^)**	SD^*^
3% BE	C^1*^	7.23^a^	0.06	7.23^a^	0.06
	C^2*^	7.35^a^	0.01	9.10^b^	0.03
	60	7.23^a^	0.14	8.90^b^	0.09
	120	6.70^b^	0.23	8.60^b^	0.09
	300	5.07^c^	0.35	7.20^a^	0.84
12% BE	C^1*^	7.26^a^	0.10	7.26^a^	0.10
	C^2*^	7.43^d^	0.02	9.63^c^	0.05
	60	7.32^d^	0.19	9.00^d^	0.07
	120	7.54^d^	0.19	9.16^cd^	0.08
	300	6.18^e^	0.19	7.93^e^	0.49
PBS	C^1*^	7.29^a^	0.04	7.29^a^	0.04
	C^2*^	7.08^g^	0.01	6.84^f^	0.03
	60	ND^*h^	–	ND^*g^	–
	120	ND^*h^	–	ND^*g^	–
	300	ND^*h^	–	ND^*g^	–

SD^*^, standard deviation; ND^*^, non-detectable; C^1*^, Control without storage; C^2*^, Control with storage. Different letters indicate a significant difference at the 0.05 level between media and treatment times.

**Table 3:** *****S. aureus*** NCTC1803 planktonic inactivation efficacy at 80 kV_RMS_ with different media and post-treatment storage conditions**.

**Media**	**Plasma treatment time (s)**	**Storage condition**			
		**4**°**C storage**		**RT storage**	
		**Cell density (Log_10_ CFU ml^−1^)**	SD^*^	**Cell density (Log_10_ CFU ml^−1^)**	SD^*^
3% BE	C^1*^	7.29^a^	0.04	6.03^a^	0.46
	C^2*^	6.63^b^	0.26	8.87^b^	0.01
	60	5.71^c^	0.34	8.08^bc^	0.17
	120	5.79^c^	0.17	7.98^c^	0.31
	300	5.02^d^	0.33	7.03^d^	0.58
12% BE	C^1*^	7.17^e^	0.06	6.30^a^	0.14
	C^2*^	6.52^b^	0.09	9.01^e^	0.14
	60	6.33^f^	0.33	8.83^ef^	0.06
	120	6.35^f^	0.22	8.76^f^	0.07
	300	5.76^g^	0.05	8.43^g^	0.09
PBS	C^1*^	7.40^ah^	0.07	6.41^a^	0.18
	C^2*^	6.37^b^	0.01	6.52^a^	0.19
	60	ND^*i^	–	ND^*h^	–
	120	ND^*i^	–	ND^*h^	–
	300	ND^*i^	–	ND^*h^	–

SD^*^, standard deviation; ND^*^, non-detectable; C^1*^, Control without storage; C^2*^, Control with storage. Different letters indicate a significant difference at the 0.05 level between media and treatment times.

**Figure 3:**
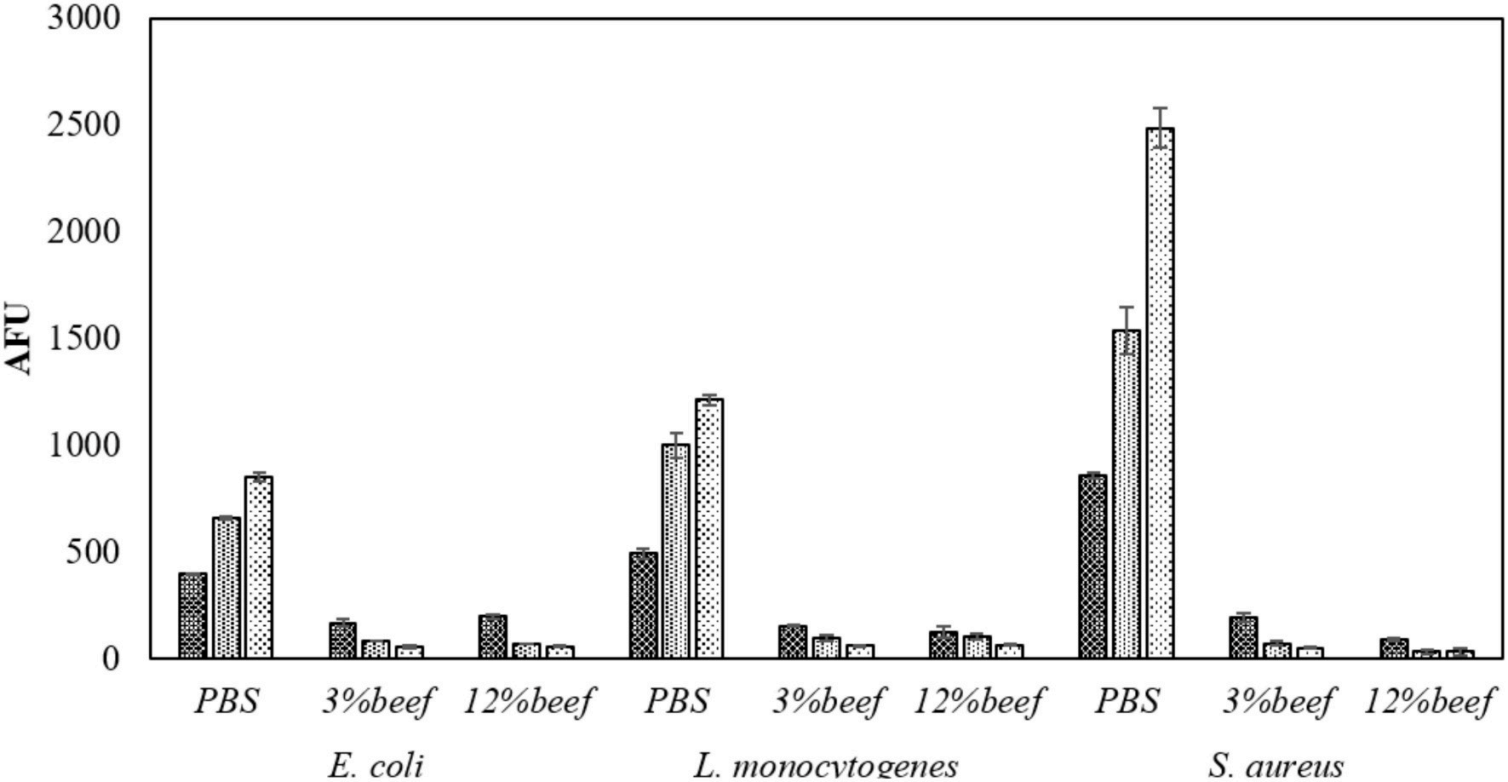
**Media associated intracellular ROS density assay by DCFH DA**. Samples in PBS, 3% BE and 12% BE were treated for 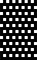 60 s, 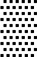 120 s, and 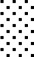 300 s at 80 kV_RMS_ and analyzed without post-treatment storage.

In PBS where there was little or no media based interference with the reactive species generated, all populations were reduced to non-detectable levels by 60 s treatments at both storage temperatures. Inactivation in both 3 and 12% BE was strongly reduced compared to treatment in PBS and required extended treatment times of 120 and 300 s to achieve any significant effects, which did not exceed 2 log total reduction (Tables 1–3). In Figure 3, the detected ROS levels from BE at either concentration were much lower than those from PBS and actually decreased with treatment time. Again, *S. aureus* suspensions had the highest ROS density among all three strains for all treatment times, and *L. monocytogenes* had slightly higher levels than *E. coli*.

At room temperature, control groups of all bacteria in BE increased by 1.87–2.84 log cycles during 24 h storage and populations in treated samples exceeded the starting cell concentrations after storage (Tables 1–3). However, at 4°C storage, no significant growth of bacteria occurred during the storage period and the inactivation achieved with ACP treatment was maintained.

### Modeling microbial recovery

*N*_*turb*_ of three strains were determined and tabulated in Table 4 before ACP treatment. Table 5 represents the results of μ_*max*_ with different ACP treatment times for the three bacteria. An overall decrease of the bacterial growth rate was observed for extended treatment times.

**Table 4:** *****N***_***turb***_ from the calibration curves**.

**Microorganism**	***N*_*turb*_ from calibration curve**	***N*_*turb*_ experimental data**
*E. coli* NCTC 12900	7.89	7.05
*L. monocytogenes* NCTC 11994	8.39	8.62
*S. aureus* ATCC 1803	8.13	7.04

**Table 5:** **Kinetic parameters of the bacteria after different ACP treatment times at 80 kV_***RMS***_ and 24 h storage at 4°C**.

**Microorganisms**	**Treatment time (s)**	***μ_max_* (h^−1^)**	SD^*^
*E. coli* NCTC 12900	C	1.26	0.55
	60	1.09	0.23
	120	0.59	0.20
	300	0.21	0.04
*L. monocytogenes* NCTC 11994	C	0.35	0.07
	60	0.17	0.09
	120	0.28	0.30
	300	0.17	0.01
*S. aureus* NCTC 1803	C	0.86	0.29
	60	0.46	0.16
	120	0.46	0.11
	300	0.31	0.34

SD^*^, standard deviation.

### Effect of different media and gas composition on ACP inactivation efficacy

As before, the inactivation efficacy increased with treatment time in PBS (Table 6), where all strains were undetectable after 60 s treatment in air. *E. coli* and *L. monocytogenes* were reduced below the detection limit after 30 s treatment in high oxygen gases, which was achieved with 60 s treatment in air. *S. aureus* had the highest resistance to ACP and was reduced from 6.88 ± 0.04 log cycles to 5.74 ± 0.19, 2.51 ± 0.45 and 1.71 ± 0.36 log cycles in 30% CO_2_ + 70% N_2_, air and 30% CO_2_+70% O_2_ respectively after 30 s treatment (Table 6, *p* < 0.05).

**Table 6:** **Planktonic inactivation efficacy at 80 kV_**RMS**_ in MAP gases in PBS with 24 h storage at 4°C**.

**Gases**	**Plasma treatment time (s)**	**Microorganisms**					
		***E. coli*** **NCTC12900**		***L. monocytogenes*** **NCTC11994**		***S. aureus*** **NCTC1803**	
		**Cell density (Log_10_ CFU ml^−1^)**	SD^*^	**Cell density (Log_10_ CFU ml^−1^)**	SD^*^	**Cell density (Log_10_ CFU ml^−1^)**	SD^*^
1	C^1*^	7.16^a^	0.07	7.51^a^	0.11	6.88^a^	0.04
	C^2*^	6.57^b^	0.24	6.93^b^	0.40	6.52^b^	0.10
	15	6.87^c^	0.09	6.48^c^	0.09	6.21^c^	0.11
	30	5.87^d^	0.03	5.83^d^	0.06	5.74^d^	0.19
	60	5.74^d^	0.05	5.21^e^	0.10	5.39^e^	0.10
2	C^1*^	7.16^a^	0.07	7.51^a^	0.11	6.88^a^	0.04
	C^2*^	6.99^ab^	0.07	6.93^b^	0.40	6.78^a^	0.08
	15	6.50^b^	0.23	6.55^bc^	0.13	5.47^c^	0.40
	30	2.98^e^	0.48	0.92^f^	0.09	2.51^f^	0.45
	60	0.00^f^	0.00	0.00^g^	0.00	0.00^g^	0.00
3	C^1*^	7.16^a^	0.07	7.51^a^	0.11	6.88^a^	0.04
	C^2*^	6.81^ab^	0.10	6.05^h^	0.17	6.30^a^	0.08
	15	6.57^b^	0.08	5.79^d^	0.02	5.11^c^	1.08
	30	ND^*f^	–	ND^*g^	–	1.71^h^	0.36
	60	ND^*f^	–	ND^*g^	–	ND^*g^	–

SD^*^, standard deviation; ND^*^, non-detectable; C^1*^, Control without storage; C^2*^, Control with storage. Different letters indicate a significant difference at the 0.05 level between gas mix and treatment times. Gases: 1 30% CO_2_ + 70% N_2_; 2 Air; 3 30% CO_2_+70% O_2_.

**Figure 4:**
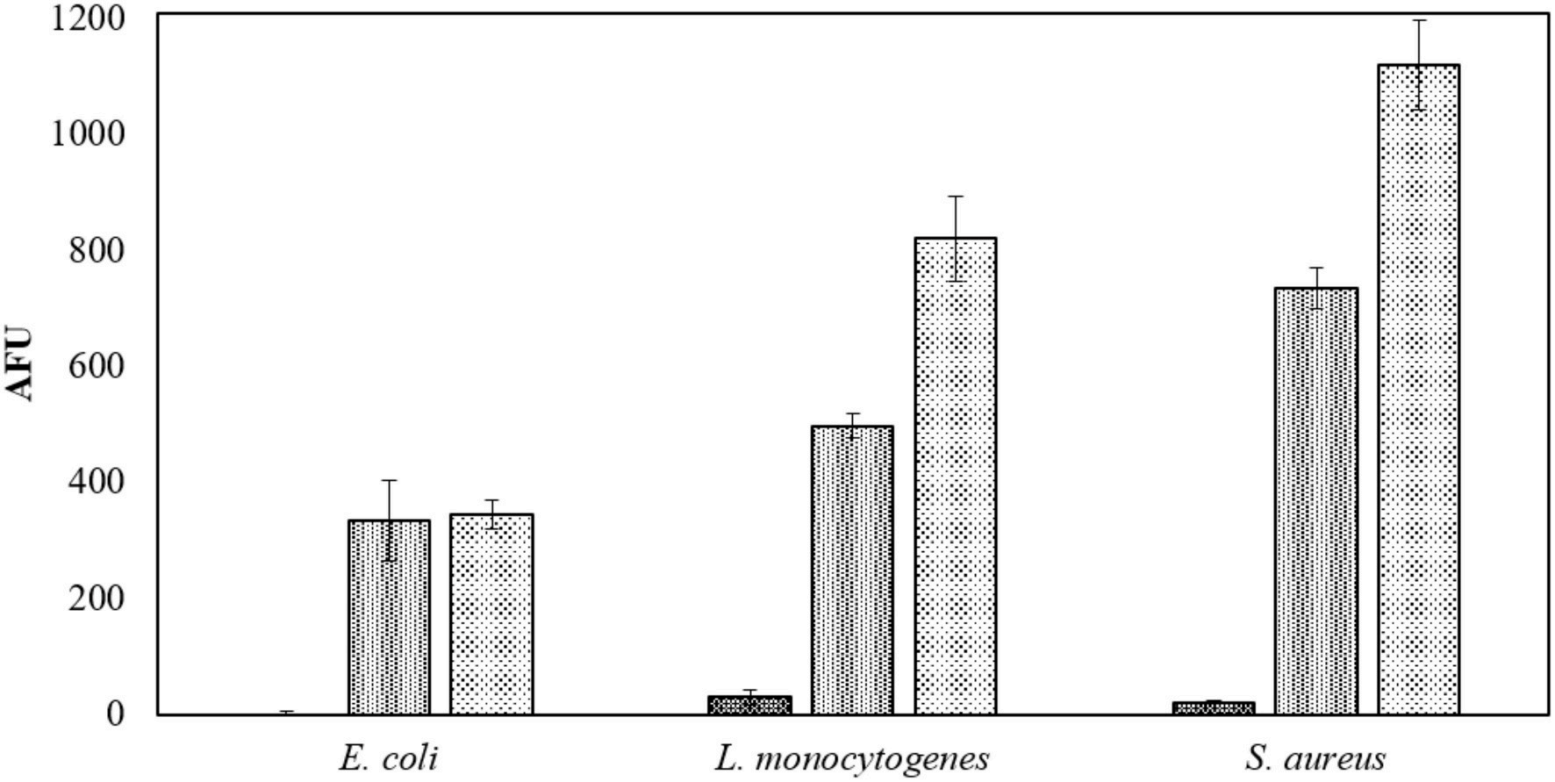
**Applied gases associated intracellular ROS density assay by DCFH DA**. Samples in PBS treated for 60 s at 80 kV_RMS_ in applied gases: 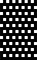 30% CO_2_+70% N_2_; 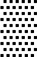 Air and 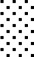 30% CO_2_+70% O_2_ and analyzed without post-treatment storage.

The beef extract maintained a strong protective effect against ACP efficacy in the modified atmospheres. One minute of ACP treatment resulted in up to 7 log reduction in PBS samples, while a maximum reduction of 2.2 log was obtained with BE samples and extended treatment times of 300 s (Tables 6, 7). In this study, in-package ozone levels were measured as an indicator of the overall ROS concentration in gas phase. Both ozone levels and detected ROS concentrations in liquid were dependent on the gas composition, where ROS were barely detected in 30% CO_2_+70% N_2_ samples and no ozone was measured inside these packages (Figure 4, Table 8).

**Table 7:** **Planktonic inactivation efficacy at 80 kV_**RMS**_ in MAP gases in BE with 24 h storage at 4°C**.

**Gases**	**Plasma treatment time (s)**	**Microorganisms**					
		***E. coli*** **NCTC12900**		***L. monocytogenes*** **NCTC11994**		***S. aureus*** **NCTC1803**	
		**Cell density (Log_10_ CFU ml^−1^)**	SD^*^	**Cell density (Log_10_ CFU ml^−1^)**	SD^*^	**Cell density (Log_10_ CFU ml^−1^)**	SD^*^
1	C^1*^	6.78^a^	0.03	7.17^a^	0.09	6.63^a^	0.07
	C^2*^	7.18^ce^	0.08	7.56^c^	0.08	7.14^b^	0.16
	60	7.64^b^	0.13	7.28^ab^	0.12	6.22^c^	0.07
	120	7.49^bc^	0.24	7.46^bc^	0.06	6.27^c^	0.07
	300	7.01^ae^	0.25	7.34^ac^	0.20	6.15^c^	0.12
2	C^1*^	6.98^de^	0.05	7.02^ef^	0.06	6.93^a^	0.29
	C^2*^	7.15^ce^	0.06	7.56^c^	0.08	7.23^b^	0.05
	60	6.87^df^	0.06	6.98^e^	0.04	6.73^d^	0.12
	120	6.74^f^	0.10	7.18^f^	0.05	6.73^d^	0.06
	300	6.55^g^	0.12	6.78^d^	0.14	5.79^c^	0.29
3	C^1*^	6.98^de^	0.01	6.99^e^	0.03	6.81^a^	0.06
	C^2*^	7.23^ce^	0.05	7.56^c^	0.08	7.17^b^	0.19
	60	6.47^h^	0.14	6.55^h^	0.22	5.85^c^	0.35
	120	5.93^i^	0.11	5.97^g^	0.03	5.54^e^	0.13
	300	5.49^j^	0.17	4.77^i^	0.05	4.63^f^	0.11

SD^*^, standard deviation; C^1*^, Control without storage; C^2*^, Control with storage. Different letters indicate a significant difference at the 0.05 level between gas mix and treatment times. Gases: 1 30% CO_2_+70% N_2_; 2 Air; 3 30% CO_2_+70% O_2_.

**Table 8:** **In-package ozone concentration with different MAP gases after different ACP treatment at 80 kV_**RMS**_**.

**Plasma treatment time (s)**	**Ozone concentration (ppm)**					
	**30% CO_2_+70% N_2_**	SD^*^	**Air**	SD^*^	**30% CO_2_+70% O_2_**	SD^*^
15	ND^*^	ND^*^	533	231	1280	179
30	ND^*^	ND^*^	1067	493	5400	849
60	ND^*^	ND^*^	2450	496	12050	2375
120	ND^*^	ND^*^	2814	1619	12240	1951
300	ND^*^	ND^*^	2950	191	16480	2305

SD^*^, standard deviation; ND^*^, non-detectable.

## Discussion

In order to evaluate the optimal conditions for antimicrobial efficacy of ACP treatment, process and system parameters including applied voltages, treatment time and MAP gas mixtures were investigated in tandem with storage conditions and treatment media reflecting real food complexity.

Increasing voltage levels generally resulted in higher inactivation of biofilms, reaching non-detectable levels in some cases (Figures 1A–C). The applied power determines the input energy of discharge, which leads to different amounts of reactive species generated and inactivation levels (Tang et al., 2011; Pankaj et al., 2013). The detected ROS levels increased along with the applied voltages, indicating the reason for faster inactivation at higher voltages (Figure 2). Treatment time also governs input energy of ACP and influences microbial inactivation by generating time-dependent amounts of reactive species. Similar trends were observed in previous studies on the inactivation efficacy correlated to voltage and treatment time (Fernández et al., 2013; Han et al., 2014; Niemira et al., 2014). These results confirmed that high voltage ACP significantly reduced key meat pathogens in a nutrient rich environment and in an attached state, and that when the highest voltage level was applied, there was no further advantage incurred with extending duration of treatment beyond 60 s, which is of relevance when retention of other fresh quality characteristics is required (Tables 1–3).

**Figure 5:**
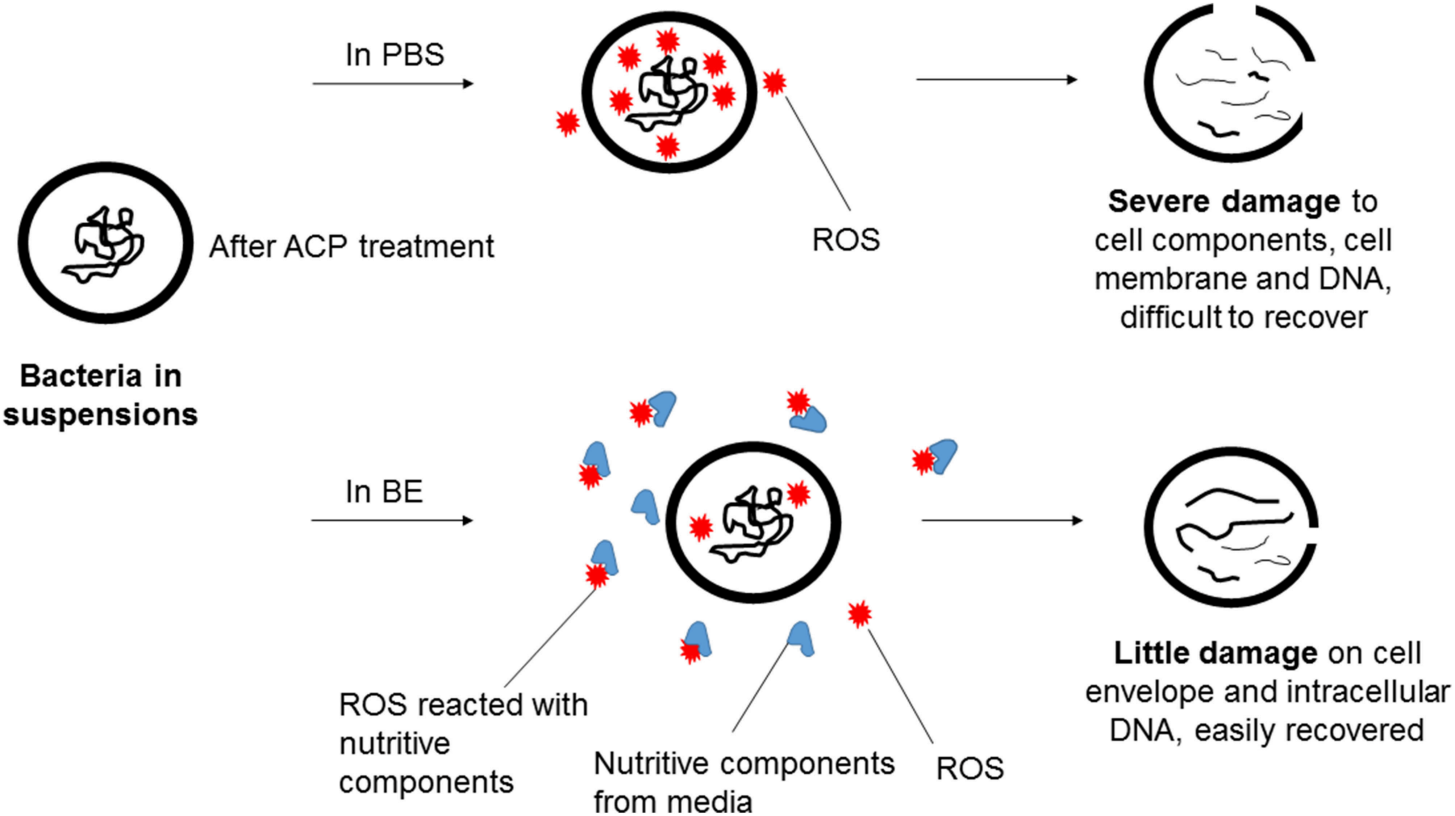
**Proposed mechanism of action of ACP generated ROS in bacteria suspensions of PBS and BE**.

Three pathogens of concern were evaluated in this study, with interesting trends emerging. Some comparison of ACP inactivation effects on different bacteria has been previously reported (Ermolaeva et al., 2011; Klämpfl et al., 2012; Liang et al., 2012). In the current study, the three bacteria displayed distinct differences in the detected ROS concentrations in PBS suspensions, although similar inactivation levels were achieved with the same treatment parameters (Figure 3). The different mechanisms of action of reactive species result from different cell envelope structures, which reflects the content of lipopolysaccharide and peptidoglycan in Gram negative and positive bacteria (Han et al., 2015). The ROS concentration detected revealed the ACP induced damage to target cells with interaction with the target cells and treatment media observed. Different inactivation efficacies were achieved using PBS and BE, where strong protective effects were observed with the BE media (Tables 1–3). Because inactivation under the detection limit was not achieved, the high nutrient content in the BE enabled regrowth after treatment, particularly under temperature abuse conditions. Assessment of ROS showed that very low concentrations of ROS were available for contact with cells treated in the BE media (Figure 3) as components of the meat medium such as proteins are likely to scavenge many of the ACP generated reactive species and thus pose a protective effect against the antimicrobial action of the reactive species (Figure 5). However, there were minimal effects of BE percentage in terms of protection and recovery of the cells, and the presence of even 3% BE indicated that the longer treatment time at the higher voltage level would be required to overcome the strong challenge to inactivation efficacy. Additionally, the results highlighted the importance of storage condition, with obvious difference from both control and treated samples under refrigerated and abuse temperature (Table 6). Refrigerated storage condition maintained the control samples at similar levels, while abuse temperature enhanced the growth significantly (*p* < 0.05). After ACP treatment, storage at 4°C maintained population densities at the reduced level, while the recovery at 37°C nullified the microbicidal effect. To assess the effect of ACP treatment following storage, a cell recovery modeling study was performed. The effect of environmental factors on the lag phase and growth rate of foodborne pathogens has been widely reported, including environmental pH, osmolality, temperature and sterilization treatment (Mackey and Derrick, 1982; Robinson et al., 1998; Mellefont et al., 2003). In our study, the μ_*max*_ was found to be ACP treatment time dependent, with *E. coli* showing more significant changes of μ_*max*_ than the Gram positive bacteria. The cell damage caused by ACP treatment resulted in a longer recovery time during refrigerated storage. A similar trend is reported in Millan Sango et al. (2015) for *E. coli*. With this effect of temperature in the cell recovery model, refrigerated storage is recommended in combination with ACP treatment in developing industrial applications.

MAP is commonly used in the packaging of meat products to extend product quality and shelf-life. The efficacy of ACP treatment for inactivation of meat contaminants was therefore investigated not only in air but also in two modified atmospheres commonly used for packed meat products. The highest inactivation levels were achieved with high oxygen content gases (Tables 6, 7). The influence of gas composition on ACP treatment has been reported, where oxygen percentage played the most important role (Laroussi and Leipold, 2004; Han et al., 2014). At the same time, the generation of RNS has also been observed in air plasma (Price et al., 2013; Ziuzina et al., 2013; Moiseev et al., 2014; Jayasena et al., 2015), where joint effects of RNS with ROS lead to higher inactivation than either group alone (Boxhammer et al., 2012). As the main mechanism of the joint effect, peroxynitrites have been fully discussed by ACP researchers as effective microbicidal species, generated through the secondary reactions of ACP generated ROS and RNS (Brisset and Hnatiuc, 2012; Lukes et al., 2014; Arjunan et al., 2015). Therefore, the use of a MAP gas combination that would mitigate the production of either ROS or RNS may not yield the best antimicrobial efficacy overall. A complete analysis of ROS and RNS generation and kinetics with this DBD system is detailed in Moiseev et al. (2014). A higher content of oxygen in MAP gas in this study led to a higher level of ROS generated, which improved the inactivation effect (Tables 6–8, Figure 4). The effect of ozone treatment of food quality has been widely evaluated, where the presence of ozone has promising decontamination effect for food product (Pérez et al., 1999; An et al., 2007). However, it could potentially damage food components, especially antioxidants in fresh-cut fruits and vegetables (Rico et al., 2007). Therefore, non-oxygen MAP gas might be an alternative choice for oxidative sensitive processing targets of ACP.

In this study, three meat pathogens were evaluated. *S. aureus* is well-known as a multi-resistant bacteria, however, its strong resistance was only observed in biofilm results here, with similar reductions to *L. monocytogenes* and *E. coli* in liquid (Tables 1–6). *L. monocytogenes* showed higher sensitivity than *E. coli* and *S. aureus* in PBS but equivalent survival in BE media, which indicates the more critical role of the nutrient environment and availability on protection or recovery. Among the parameters studied, the target properties, including planktonic or biofilm and nutritive composition in media, were crucial for ACP inactivation. While a strong interactive effect of treatment time and gas composition was observed from PBS planktonic models, the influence of these parameters were minimized by the protection of surface properties.

## Conclusion

Overall, the process and system parameters of ACP treatments were found to govern the inactivation efficacy by generating different amounts of reactive species. The nutrient content available at meat surfaces could provide protective effects against ACP treatment with reduced inactivation levels due to the decreased ROS levels. Moreover, Gram positive and negative bacteria showed significant differences in the quantities of ROS detected, implying different damaging patterns, although ACP had similar antimicrobial efficacy against both. Additionally, maintaining refrigerated storage temperature will maintain the microbial reductions achieved by ACP treatment. These results indicate both the potential and the limitations of the use of ACP for inactivation of microbiological challenges to meat safety and can help to develop and optimize treatment strategies. The further development of HV ACP for meat decontamination can be determined according to the characteristics of the biological target, the matrix and/or surface characteristics in addition to equipment design.

## Author contributions

PB, LH, VV, PC, and DB conceived and designed the experiments; LH, DZ, DS, CH, AP performed the experiments; PB, LH, DB, VV, DS wrote the paper.

### Conflict of interest statement

The authors declare that the research was conducted in the absence of any commercial or financial relationships that could be construed as a potential conflict of interest.
